# Nano-emulsion of garlic oil extracts: development, characterization and nematicidal efficacy against *Meloidogyne incognita* on tomato

**DOI:** 10.1038/s41598-026-57476-4

**Published:** 2026-06-26

**Authors:** Mohamed A. Radwan, Hamdy R. Soltan, Gomaa M. Gomaa, Mohamed H. Khalifa

**Affiliations:** Department of Pesticide Chemistry and Technology, Faculty of Agriculture, University of Alexandria, El-Shatby 21545, Alexandria, Egypt

**Keywords:** Nematicidal activity, Nanoemulsion, GC-MS, Garlic extracts, Nanotechnology, Root-knot nematodes, Biochemistry, Biological techniques, Chemistry, Environmental sciences, Plant sciences

## Abstract

The root-knot nematode, *Meloidogyne incognita*, causes serious economic losses in agricultural production worldwide. Although traditional nematicides have proven effective, their utility is becoming increasingly restricted by regulations due to risks to ecosystems and public health. Consequently, plant-based extracts are gaining popularity as an eco-friendly alternative for nematode control. However, the‍‌‍‍‌ limitations of using plant extracts necessitate the development of novel ‍‌‍‍‌formulations. Therefore, garlic oil was extracted with ethanol or ethyl acetate and analyzed for composition by GC-MS. It was developed as a nanoemulsion, characterized and then tested for use as an alternative strategy to manage *M. incognita* infecting tomato plants. The results showed that garlic oil extracted with ethanol contained a high concentration of bis-(2-diethyl aminoethyl) trisulfide (23.74%), methyl propyl tetrasulfide (18.85%), and isopropyl tert-butyl disulfide (10.93%). 1,3-dipentyltrisulfane (74.18%) and 2,4-dimethylphenyl 2-naphthyl sulfone (15.52%) were the main constituents in garlic oil extracted with ethyl acetate. The resulting six nanoemulsion formulations were prepared using the high-energy emulsification method and were characterized by tiny droplets (≤ 138 nm), the polydispersity index (PDI) (≤ 0.65), zeta potential values in the range of 10.2–20.3 mV, and spherical morphology. These formulations maintained stable for 16 weeks when stored at 25 °C. Meanwhile, soil drenching with the nanoemulsions effectively managed *M. incognita* by reducing the formation of root galls and 2nd juveniles in the soil and promoting tomato growth, particularly the Tween 20 and Tween 80 formulations, both of which were ethanol-based and demonstrated superior nematicidal effects compared to ethyl acetate extract as well as the tested nematicides; cadusafos and oxamyl. Overall, the garlic oil nanoemulsion formulations developed in our study appear to be promising candidates for the development of novel and green nematicide formulations for managing *M. incognita* associated with tomatoes in sustainable agriculture.

## Introduction

Plant-parasitic nematodes (PPNs) are animal pests that pose the greatest sever to global agricultural production. They cause significant economic losses and impact the sustainability of agricultural ecosystems. Approximately 4,100 species of PPNs have been identified, and their annual yield losses worldwide exceed US$173 billion^1^, reducing crop quality and yield. Tomato (*Solanum lycopersicum* L.) is an important vegetable crop grown around the world, and its yield is affected by a variety of plant pathogens, including PPNs, the most damaging of which is the root-knot nematode (*Meloidogyne* spp. RKNs). Due to its high reproduction rate, short generation time, and ability to damage a wide variety of host plants, *Meloidogyne incognita* (Tylenchida: Meloidogynidae), a prominent member of the RKN, poses a threat to almost all vegetable crops, especially in tropical regions. Tomato yields in Egypt are severely threatened by RKNs, with estimated annual losses of about US$80 billion^2,3^. RKNs are sedentary endoparasites that damage the integrity of the root system by forming giant cells, severely restricting nutrient absorption, stunting growth, causing chlorosis and wilting, which reduces crop yield and quality^4^. Furthermore, these nematodes often facilitate secondary infections caused by fungal, bacterial, and viral pathogens, leading to complex disease interactions that worsen host plant diseases^5^. Considering the above, the most difficult challenge is to keep nematode populations below economically damaging levels through a nematode management program.

Various approaches to nematode management have been adopted, including cultural, physical, biological, resistant plant varieties, organic soil amendments, soil solarization, botanical extracts and chemical methods^6^. Historically, PPNs management has largely relied on synthetic nematicides, which are crucial to safeguarding the world’s food supply. Nevertheless, the significant risks associated with synthetic nematicides to the environment, human health, and soil health cannot be ignored^7^. As a result, regulatory measures, including the European Union’s Directive 91/414/EEC and its later updates, have imposed strict limitations or complete prohibitions on many traditional nematicides^8^. These regulatory shifts have intensified the critical need for environmentally friendly alternatives that are compatible with sustainable agricultural systems^9^. Among the available options, plant extracts have attracted significant attention because of their abundance of bioactive secondary metabolites^10^. Unlike synthetic nematicides, plant extracts are generally biodegradable, readily available locally, cost-effective, less harmful to non-target organisms, and better suited to agricultural ecosystems.

Garlic, scientifically known as *Allium sativum*L., is a globally cultivated plant and highly popular species within its genus. For centuries, it has been valued as a food ingredient, spice, and medicinal herb. The plant’s therapeutic effects are associated with a wide range of biological properties, including antibacterial, cardiovascular, anti-inflammatory, anticancer, antidiabetic, anti-Alzheimer’s, and antioxidant activities^11,12^. These biological and medicinal effects of garlic are primarily due to its high content of sulfur-containing compounds such as alliin, ajoene, allicin, vinyldithiin, sulfides, allylcysteine and some polysulfanes (diallysulfide, diallyldisulfide, diallyltrisulfide, diallyltetrasulfide), which are some of the components isolated from *A. sativum*extracts. Garlic extracts, obtained using organic solvents of varying polarity, are rich in a variety of natural bioactive compounds, resulting in differences in biological activity^13^. Due to their rich content of organosulfur compounds, garlic extracts and their essential oils exhibit broad-spectrum activity against a variety of agricultural pests, including insects^14,15^, fungi^16^, bacteria^17^, mollusks^18^, and plant nematode^19–21^. Their efficacy is mostly because of those compounds, which disrupt pest biology and decrease pest resistance *via* multiple mechanisms of action. Although garlic oil extract is safe for consumers^22^, its natural physical and chemical properties create significant obstacles for field application. The main issues that have to be addressed before garlic oil extract can be used as a pest control tool are its high volatility, phytotoxicity, low water solubility, rapid decomposition, and high flammability^23^.

The constraints of garlic oil extract present challenges in assessing their potential for creating effective biocides against PPNs. A further significant factor is the volatile character of oils and garlic’s tendency to oxidize its sulfur compounds^24^. Because of these characteristics, garlic oil extract likely persists in the soil for a shorter duration of time compared to non-fumigant nematicides. Consequently, the majority of nematodes may not encounter the oil within the soil, rendering nematode management ineffective. A more advantageous strategy for reducing nematode populations would involve applying garlic oil extract as a fumigant biocide to the soil prior to planting. Additional studies are required to address the uncertainties surrounding the efficacy of garlic oil extract against PPNs. Nevertheless, such compounds frequently exhibit limited long-term stability, unregulated volatility, or very low bioavailability, necessitating innovative encapsulation methods. Recent research has concentrated on nano-emulsion technologies, as they are ideally suited for developing novel products^25^.

The application of nano-emulsion technology offers a means to prevent degradation by establishing an encapsulation system for active ingredients. This approach also enhances the bioaccessibility, stability, and solubility of the compounds involved^26^. Fundamentally, nano-emulsions are formed from two immiscible liquids that do not blend into a uniform solution. They are categorized based on the continuous phase: as water-in-oil (W/O) or as nano-scale oil droplets dispersed in water (O/W). In these systems, one liquid is dispersed as minute spherical droplets within the other^27^. The formulation of nano-emulsions typically requires several components: an oil phase, an aqueous phase, an emulsifier, and potentially a co-surfactant. The stability of the resulting emulsion can be influenced by the processing technique; as different preparation methods can produce droplets within varying size ranges. A compelling method for administering natural oils involves nano-emulsions, where the oils are encapsulated within nanosized micelles measuring 20 to 200 nm. These systems possess distinct properties, including their minute dimensions, enhanced surface area, and stability, which can boost the biological effects and potency of essential oils.

Garlic oil extract has proven to be a reliable source of several biologically active compounds, making it useful as a natural pesticide against various pests, including PPNs. Despite the acknowledged promise of garlic oil extract for managing *Meloidogyne* spp. is well-documented^19,28–31^, a considerable information void persists concerning the formulation of its nanoemulsion version and its impact as a nematicide^32,33^. Consequently, the discovery and development of natural pesticides for pest control is critically important. Our study’s goal was to develop, characterize, and assess the nematicidal efficacy of various garlic oil extracts-nanoemulsions (GOE-NEs)against *M. incognita*. Therefore, this study focused on the development, characterization, and evaluation of garlic oil nanoemulsions for managing *Meloidogyne incognita* infestations in tomatoes under greenhouse conditions. Our study is important as it will highlight the role of nanoemulsion technology in improving the efficiency of garlic oil extracts and help in developing new sustainable root-knot nematode management by reducing the dependence on synthetic nematicides.

## Materials and methods

### Chemicals and nematicides

The emulsifiers were supplied by Sigma-Aldrich (Steinheim, Germany), including polyoxyethylene sorbitan monooleate (Tween 80), sorbitan fatty acid esters (Span 80), and the supplier of the polysorbate 20 (Tween 20) was VWR International, located at 201 Rue Carnot F-94,126 Fontenay/Bois, France. The reagents and solvents were of reagent standard quality and were procured from local scientific suppliers in Egypt. FMC Corporation USA supplied the nematicide cadusafos (Rugby^®^ 10% G), while Du Pont Egypt provided the nematicide oxamyl (Vydate^®^ 24% SL).

### Preparation of garlic oil extracts using organic solvents

Garlic cloves were collected from local markets in Alexandria, Egypt. A 500 g garlic bulb was peeled, cleaned, and sliced into cloves. A total of 400 g of peeled cloves were finely chopped and immersed in 500 mL of ethanol or ethyl acetate for 48 h to extract the supernatant. To recover the oil from the remaining garlic, three more solvents were used. By distilling the solvent off the mixture of extracts at 40 °C, the oil was obtained. Centrifugation was performed on the oil after it had been mixed with 20 volumes of redistilled petroleum ether (40–60 °C). The oil portion soluble in petroleum ether was extracted by distilling the solvent at 60 °C after the separation of the clear supernatant^34^.

### Gas liquid chromatograph-mass spectrometer analysis

To examine the chemical composition of garlic oil extracts, a gas-liquid chromatograph connected with MS (Shimadzu 2010) using a capillary column OV17X was used.

AHP GC-mass selective detector (5971B MSD) was attached to a Hewlett-Packard 5890 (series II) gas chromatograph that had been modified for a glass capillary column. Gas chromatography was used to evaluate hydrocarbons and the methyl esters of fatty acids using two capillary-linked columns, as outlined in earlier work^35^, and a 10-m HP-5 column with an internal diameter of 0.32 mm, and a film thickness of 0.25 mm, connected to a second capillary column, an RTX-1701 (Restek, PA, USA) with a length of 30 m, an internal diameter of 0.32 mm, and a film thickness of 0.25 mm. A third capillary column, an HP-FFAP column with a length of 30 m, an internal diameter of 0.32 mm, and a film thickness of 0.25 mm, was also connected. The GC oven was programmed as follows: 40 °C for 2 min, ramped to 300 °C at 2 °C/min, and held at 300 °C for 20 min. The injector temperature was maintained at 180 °C (splitless mode). The flow rate of the carrier gas (helium) was 25 cm/s. The MS detector was operated at 194 °C with an ionization energy of 70 eV. The scan range was 30–650 m/z at the scan rate was 0.9 s^− 1^. The solvent delay time was 10 min. Before injection, the oil samples were diluted by adding 20 µL to 1 mL of n-hexane. Organosulfur compounds were detected using the mass spectral library from the National Institute of Standards and Technology (NIST).

### Nano-emulsion preparation of garlic oil extracts

High-energy emulsification procedures use stirrers, homogenizers, and ultrasonic devices to create nano-emulsions. A garlic oil nano-emulsion was developed and characterised in this study. A high level of mechanical energy is applied to garlic oil prior to processing to generate stronger turbulent forces, which break down large molecules into smaller particles. The surface of the garlic oil was thereby enhanced for surfactant adsorption. The disruptive force that is produced by ultrasonic instruments is what enables the production of nano-emulsions. The use of a high-energy method enables us to control the particle size and composition of formulations, as well as their stability and rheology. Nano-emulsions can be controlled with this process. The final optimal conditions of garlic oil extract were achieved by using several different formulations, including the organic phase, surfactant sonication pulses, and sonication strength.

### Method 1 (formulation code F1 using Tween 20)

The first technique utilised to create an oil/water nano-emulsion (O/W) containing garlic oil (formulation code F1) included distilled water, oil extract, and Tween 20, a non-ionic emulsifier. To form the organic phase (oil and emulsifier), garlic oil and the emulsifier were mixed in various ratios (1:1, 1:1.1, and 1:1.2 W/W) using a magnetic stirrer. To create the primary emulsion, the organic phase was then gradually added to the distilled water, drop by drop. Employing a 7500 W ultrasonic probe running at 20 kHz, the primary emulsion was transformed into a nano-emulsion. Following the addition of the organic phase to the distillate, sonication was carried out for 15, 25, and 35 min. Throughout the process, ice was utilised to cool, and a probe on the sonicator provided energy^36^.

### Method 2 (formulation code F2 using Tween 80)

Using a magnetic stirrer, garlic oil and Tween 80 as a non-ionic emulsifier were combined for five minutes at 500 rpm to create the second method of making garlic oil nano-emulsion (formulation code F2). The distilled water containing 0.8% citric acid was then gradually mixed with the combined oil phase (emulsifier and garlic extract mixture) for fifteen minutes at a speed of seven hundred revolutions per minute. Once that was done, the pre-mixed formulations were placed in an ultrasonic water bath set at 750 W and 20 kHz, and they were left there for 15, 25, and 35 min. In the study, the ratios of surfactant to oil varied from 1:1 to 1:1.1 to 1:1.2^37,38^.

### Method 3 (formulation code F3 using Tween 80 and Span 80 blend)

To achieve a stable formulation during storage, Long et al.^39^ emphasized the importance of attaining the optimal hydrophilic-lipophilic balance (HLB) for garlic oil. An emulsifier mixture (Smix) with an HLB value of 14 can be produced by combining Tween 80 and Span 80 in a 9:1 weight-to-weight ratio to reach this goal. Garlic oil and Smix were initially mixed using a magnetic stirrer in a 1:1 w/w ratio to form the organic phase. After that, the organic phase and the water phase, which consisted just of distilled water, were equally combined to form the first emulsion. The emulsion was then subjected to an ultrasonic processor delivering an output power of 750 W. Operating at 80% capacity, the processor utilized a 6 mm titanium alloy probe and applied a pulsed sequence of 5 s on and 7 s off for three minutes to prevent the samples from overheating. This process resulted in the formation of a garlic oil nano-emulsion.

## Characterization of nano-emulsions

### Stability studies

Based on the FAO/WHO Manual^40^, the garlic oil emulsifibale concentrate was subjected to 14 days of stress storage conditions: hot storage (54 ± 2 °C) and cold storage (0 °C) before formulating a nanoemulsion. This ensured that the “building blocks” of the developed nanoemulsions were robust enough to withstand high-energy processing (such as ultrasonication) and provide a long shelf life. Numerous physico-chemical and stability tests using the centrifugation, heating, cooling, and freezing cycles, as well as thermodynamics, were performed on garlic oil nano-emulsions^41,42^. Every nano-emulsion formulation underwent a centrifugation test. Following the centrifugation of formulation nano-emulsions at 10,000 rpm for 30 min at 25 °C in a Heraeus Labofuge 400R, possibilities for phase inversion were examined. This technique has been widely applied to the stability investigation of both conventional and nano-emulsions^43^. Formulations without phase separation were exposed to the heating and cooling cycles. Physical appearance was assessed throughout six cycles, with two days of chilling at 4 °C and another day of being heated at 50 °C over two days. When the separation of phases or precipitation is not evident, the formulation is said to be stable. For formulations considered stable after six cycles, frozen-thawed cycle tests were performed. Three temperatures were examined for a freeze-thaw cycle, ranging from ambient temperature to −20 °C for two days. The cycle consisted of 24 h at ambient temperature, followed by 24 h at −20 °C. To assess stability, visual appearance was observed.

### Analysis of droplet size, zeta potential, and polydispersity index

Garlic oil nano-emulsions were subjected to measurements of the mean particle size and homogeneity of the size distribution (polydispersity index, PDI) using the dynamic light scattering (DLS) method and a zeta-sizer (Nano-ZS, Malvern Instruments, Malvern, UK). The trials were carried out with a 90° angle of refraction and at 25 °C. It has a 4 mW He-Ne laser (λ = 633 nm) and non-intrusive backscatter optics (NIBS). The size of the droplet was determined by tracking the rate of change in the laser light’s intensity as the droplets moved and scattered. The translational diffusion coefficient and droplet size were determined using the Stokes-Einstein equation, assuming that spherical particles (the corresponding spheres) are in Brownian motion^26^. The zeta potential of these droplets was also measured with the same apparatus.

### Microstructure studies

A transmission electron microscope (TEM) equipped with a CCD camera, the JEM-1400 Plus electron microscope, was used to analyse the morphology of several distinct nano-formulations. The negative film was subsequently colored with a 3% phosphor tungstic acid (PTA) aqueous solution after two to three drops of nano-emulsion samples were put onto the film faces of 200 mesh formvar/carbon-coated copper grids without dilution. Filter paper was used to carefully clean away any surplus solution during each stage. A room temperature was used for the entirety of the procedure. The sample was put into the TEM for 200 kV imaging. TEM images were captured using imaging modes ranging from low magnification (LM 2100x) to high magnification (SA 145000x).

### Nematicidal evaluation of garlic oil extract-nanoemulsions

#### Collection and preparation of nematode inoculum

Root-knot nematodes belonging to the genus *Meloidogyne* spp. were gathered from infected eggplant (*Solanum melongena* L.) roots obtained from the El-Bostan region of Behera Governorate, northern Egypt. This isolated population was subsequently identified as *M. incognita* based on the diagnostic guidelines provided by Taylor and Nelscher^44^. Eggs were extracted from the infested root material using sodium hypochlorite (NaOCl) following the procedure outlined by Hussey and Barker^45^, and second-stage juveniles (J2) were collected *via*the Baermann plate method^46^.

### Greenhouse pot experiment

A greenhouse assay was carried out to evaluate the nematicidal effectiveness of six GE-NE formulations, designated F1.1, F1.2, F2.1, F2.2, F3.1and F3.2, in comparison with the commercial nematicides; cadusafos and oxamyl, in controlling *M. incognita* infecting tomato plants. Each plastic pot, 15 cm in diameter, was filled with 1 kg of autoclaved sandy loam soil. A single three-week-old tomato seedling of variety strain B was transplanted into each pot. Standard fertilization and watering procedures were followed, and the pots were allowed to sit for three days to let the seedlings acclimate to the greenhouse conditions. Every treatment was replicated six times and arranged in a randomized complete block design on a greenhouse bench, kept at 30 ± 2 °C, 63 ± 2% relative humidity, and a 14:10 light-dark cycle. Three days after transplanting, each plant was inoculated with 5000 eggs. Two days later, 1000 mg of each garlic oil nanoemulsion formulation was applied per kilogram of soil. For reference, cadusafos and oxamyl were employed as standard nematicides, each applied at a dosage of 0.02 a.i. g/kg soil. All treatments were delivered as a soil drench. Control groups consisted of untreated, uninoculated plants and untreated plants inoculated with *M. incognita*. Sixty days after inoculation, the plants were carefully removed, and the roots were cleaned of soil. Data gathered included shoot and root lengths, the fresh weights of both shoots and roots, the total number of galls on each root system, along with the population of J2 recovered from a 250 g soil sample. The extraction of J2s from the soil was conducted using the decanting and sieving technique as described in earlier descriptions.

### Statistical analysis

The Shapiro-Wilk test was used to evaluate data normality, while the Levene’s test was applied to check for homogeneity of variance. Analysis of variance was used to analyse the data (ANOVA) with the SAS software program^47^, and mean separation was conducted using Duncan’s Multiple Range Test (*p* < 0.05).

### Ethical approval and consent to participate

This article does not contain any studies with human participants or animals performed by any of the authors. The current experimental research including the collection of plant material, is complying with relevant institutional, national, and international guidelines and legislation and used for research and development.

## Results and discussion

### Chemical composition

Garlic oil extracted with ethanol or ethyl acetate used in the extraction process was the same in yield, but showed significant quantitative differences in bioactive component content. Data in Table 1 show that seventeen compounds were identified in the ethanolic extract of garlic bulb, the major compounds were bis-(2-Diethyl aminoethyl) trisulfide (23.74%), methyl propyl tetrasulfide (18.85%), isopropyl tert-butyl disulphide (10.93%), bis(1-propenyl)sulfide (6.37%), bis(1-methyl propenyl) disulphide (5.88%), dithio bis(thionoformic acid) (5.84%), while methyl − 5-methylfuryl disulphide (3.26%), 3 H-1,2-dithiole (2.97%), 1,2-dithiolane (1.48%), 1,4-dimethyl tetrasulfide (1.26%), and 2,4-diethyl-2,5-dihydro-thiophene (0.63%) as minor components. The experimental results obtained by other researchers differ from recent studies. Dehariya et al.^48^ used Soxhlet extraction method to isolate oil from garlic powder using ethanol as a solvent, and the major chemical components were diallyl disulfide (48.42%), allyl methyl trisulfide (7.27%), trisulfide, di-2-propenyl (3.46%), and diallyl sulfide (7.64%). This result differs from the results of another author^49^. In that study, the major components of garlic oil were found to be 3-vinyl-4 H-1,2-dithiine (31.89%), diallyl trisulfide (13.31%), diallyl sulfide (2.22%), diallyl disulfide (6.87%), propyl allyl disulfide (13.89%), and dimethyl disulfide (7.05%). Moreover, Bajac et al^50^. found that ethanol extract of garlic contained 4.39–4.56 µg/mL of allicin, along with small amounts of other sulfur compounds.

**Table 1:** List the ingredients of the garlic oil, extracted using ethanol or ethyl acetate, and identified by GC-MS.

NO.	Ethanol			Ethyl acetate		
	Ingredients	*R*_t_ (min)	Area (%)	Ingredients	*R*_t_ (min)	Area (%)
1	2,4-diethyl-2,5-dihydro-thiophene	4.89	0.63	diallyl disulfide	5.44	0.40
2	3 H-1,2-dithiole	5.64	2.97	methyl n-hexyl disulfide	6.37	0.70
3	3-Vinyl-[4 H]−1,2-dithiin	5.73	1.19	Bis (1-methyl propenyl) disulfide	6.3	1.07
4	Isopropyl tert-butyl disulfide	6.32	10.93	3-Vinyl-[4 H]−1,2-dithiin	16.22	2.30
5	Bis (1-methyl propenyl) disulfide	6.39	5.88	2-(ethyl thio)−3-methyl-1-butene	10.86	1.59
6	1,4-dimethyl tetra-sulfide	6.83	1.26	1,3-dipentyltrisulfane	29.93	74.18
7	1,2-dithiolane	7.82	1.48	1-allyl-3-(2-(allyl di-sulfanyl) propyl) tri-sulfane	30.62	1.61
8	dimethyl trisulfide	8.94	3.96	disulfide, (diethyl thiocarbamoyl)	33.39	2.11
9	Bis(1-propenyl)sulfide	9.77	6.37	tetra-sulfide, dihexyl	33.74	0.51
10	methyl − 5-methylfuryl disulfide	10.18	3.26	2,4-dimethylphenyl 2-naphthyl sulfone	39.65	15.52
11	2,3,5-trithiahexane 5-oxide	28.4	0.94			
12	1,2-benzenedicarboxylic acid, bis(2-butoxyethyl) ester	30.08	0.64			
13	methyl propyl tetra-sulfide	30.63	18.85			
14	1,2-diphenyl sulfonyl hydrazine	31.49	0.94			
15	diallyl pentasulfide	31.69	0.74			
16	Bis-(2-diethylaminoethyl) trisulfide	33.4	23.74			
17	Dithio bis (thiono formic acid)	33.74	5.84			

When ethyl acetate was used for garlic oil extraction, the findings presented in Table 1 revealed that the major components were 1,3-dipentyltrisulfane (74.18%) and 4,4-dimethylphenyl 2-naphthyl sulfone (15.52%), and the trace components were 3-vinyl-[4 H]−1,2-dithiine (2.3%), (diethyl thiocarbamoyl) disulfide (2.11%), 1-allyl-3-(2-(allyl disulfanyl)propyl)trisulfane (1.61%), 2-(ethylthio)−3-methyl-1-butene (1.59%), bis(1-methylpropenyl) disulfide (1.07%), methyl n-hexyl disulfide (0.7%), and diallyl disulfide (0.4%). Comparing the garlic oil constituents obtained in this investigation with those of earlier studies, quantitative differences were found. It is well known that plant species and subspecies (genetic factors) affect the yield and chemical composition of volatile compounds. Furthermore, even within the same plant, the chemical composition and yield of volatile compounds can vary depending on soil type, climate, growth stage, and others. According to Kimbaris et al.^51^, garlic volatile compounds contain highly reactive sulfur compounds that interact with various separation techniques. Ultrasonic treatment using ethyl acetate is known to minimize damage to heat-sensitive molecules, and the major components of garlic oil were identified as 2-vinyl-[4 H]−1,3-dithiine (38.1%) and 3-vinyl-[4 H]−1,2-dithiine (32.7%). In contrast, Hincapié et al.^52^ reported that the total vinyl dithiine content obtained by soaking garlic in ethanol was 15.19%. Our GC-MS results suggest a close correlation between the components of extracted garlic oil and the solvent used for extraction. Garlic oil was extracted using ethanol and ethyl acetate, which have different polarities, and exhibited distinct qualitative and quantitative differences. When the extraction solvent was changed from ethanol to ethyl acetate, the major disulfide content decreased from 20.6% to 2.17%, while the trisulfide content increased from 27.7% to 74.18%, respectively, demonstrating a significant quantitative change. Bar et al^13^. reported that solvent type affects the amount and type of bioactive compounds obtained from garlic extracts, and that the content of bioactive compounds is correlated with the biological activity of the extract.

### Development of nano-emulsion formulations

Garlic oil’s industrial utility is limited by its volatility, odor, and low water solubility. To improve its efficacy, nanoemulsion formulations are used to encapsulate the oil into smaller droplets with larger surface areas. This nanotechnology enhances stability, increases water solubility, and improves the penetration of active compounds through biological barriers, allowing for lower dosages and higher performance. Compared to conventional emulsions, nanoemulsions have a larger surface area and smaller droplet size, maximizing accessibility and efficacy by encapsulating active compounds within the dispersed phase^53^.

### Method 1 (formulation code F1 using Tween 20)

The most important factors in nanoemulsion preparation were operating parameters and formulation characteristics. High-pressure homogenization is influenced by several factors, including the type and amount of oil and emulsifier, and the weight ratio of emulsifier to cosurfactant. Various garlic oil (5%) formulations were prepared using polysorbate 20 as an emulsifier and distilled water to determine the appropriate emulsifier ratio for use in garlic oil nanoemulsions. Table 2 shows the garlic oil and emulsifier ratios ranging from 1:1 to 1:1.2 (w/w). Sonication times were also set at 15, 25, and 35 min. As shown in Table 3, various stability studies, including thermodynamic and centrifugal tests, were performed on the F1 formulations (F1-A 1–3, F1-B 1–3, and F1-C 1–3). The stability test results revealed significant variation in the formulations (F1-A 1–3, F1-B 1–3, and F1-C 1–2), which exhibited varying degrees of creaming and phase separation. Therefore, these formulations were discarded for further study.

**Table 2:** Ratios between garlic extract and emulsifier type to prepare different nano-emulsions.

Formulation code*	Garlic oil: emulsifier ratio (w/w)	Formulation ingredients (%)		
		Garlic oil	Emulsifier	Distilled water
F1-A/F2-A	1:1	5	5	90
F1-B/F2-B	1:1.1	5	5.5	89.5
F1-C/F2-C	1:1.2	5	6	89

* F1. A, B and C represent garlic extract: Tween 20 ratios.

* F2. A, B and C represent garlic extract: Tween 80 ratios.

**Table 3:** Stability tests on the nano-emulsions made from garlic extracts and emulsifier.

Sample code	Sonication Period (min.)	Centrifugation		Heating-Cooling cycle		Freeze-Thaw cycle		Result*	
		F1	F2	F1	F2	F1	F2	F1	F2
F1-A1/F2-A1	15	-	-	-	-	-	-	X	X
F1-A2/F2-A2	25	-	-	-	-	-	-	X	X
F1-A3/F2-A3	35	+	+	+	+	-	+	X	√
F1-B1/F2-B1	15	-	-	-	-	-	-	X	X
F1-B2/F2-B2	25	+	+	+	+	-	-	X	X
F1-B3/F2-B3	35	+	+	+	+	-	+	X	√
F1-C1/F2-C1	15	-	-	-	-	-	-	X	X
F1-C2/F2-C2	25	+	+	+	+	-	-	X	X
F1-C3/F2-C3	35	+	+	+	+	+	+	√	√

* x failed, √ passed.

F1. A, B and C represent garlic extract with Tween 20.

F2. A, B and C represent garlic extract with Tween 80.

Each formula exposed to sonication periods of 15 min (1), 25 min (2), and 35 min (3).

Nanoemulsions containing garlic extract and polysorbate 20 in a 1:1.2 (w/w) ratio and prepared by sonication for 35 min were found to be stable during centrifugation, heating, refrigeration, and freezing. The most stable nanoemulsion was F1-C3, which did not exhibit phase separation after 16 weeks of storage at room temperature (Table 3). The selected formulation did not exhibit sedimentation, emulsion breakdown, turbidity increase, or creaming after centrifugation, heating-cooling, or defrosting cycles. Successive thawing of the optimized formulation demonstrated excellent freeze stability. These droplets matched the HLB values of the oils^54,55^. F1-C3 was the most stable nanoemulsion, which did not exhibit phase separation. As shown in Table 4, these optimization conditions were applied to the preparation of nanoemulsion formulations of garlic oil extracted by ethanol (formulation codes F1.1, F2.1, and F3.1) and nanoemulsion formulations of garlic oil extracted by ethyl acetate (formulation codes F1.2, F2.2, and F2.3).

**Table 4:** Percentages of garlic extract, emulsifier, and distilled water used for preparing of nano-emulsions.

Formulation code*	Garlic extract: emulsifier ratio (w/w)	Formulation ingredients (%)				
		Garlic extract	Emulsifier			Distilled water
			Tween 20	Tween 80	Span 80	
F1.1	1:1.2	5	6	-	-	89
F1.2	1:1.2	5	6	-	-	89
F2.1	1:1	5	-	5	-	90
F2.2	1:1	5	-	5	-	90
F3.1	1:1	5.5	-	0.55	4.95	89
F3.2	1:1	5.5	-	0.55	4.95	89

*F1, F2, and F3 represent garlic extract nanoemulsion developed using Tween 20, Tween 80, and Tween 80 + span 80 blend, respectively.

F1.1, F2.1, and F3.1 code formulations of garlic ethanol extract.

F1.2, F2.2, and F3.2 code formulations of garlic ethyl acetate extract.

Evaluating the quality and stability of nanoemulsions primarily depends on two key factors: droplet size and the PDI. These characteristics are measured using Dynamic Light Scattering (DLS) to ensure a uniform and stable formulation. The mean value of the particle size distribution (PSD) serves as the primary indicator of the system’s physical properties and overall performance^56,57^. To assess the quality of nanoemulsion formulations, two metrics are vital: average droplet size and PDI. Droplet size directly dictates the stability, appearance, and delivery efficiency of the system. The PDI measures uniformity; a value below 0.3 signifies a stable, narrow distribution, whereas a value near 1.0 indicates high variability and potential instability^58^.

Zeta potential measures the electrical charge density of droplets and is a key predictor of nanoemulsion stability^55,57^. High zeta potential values (typically greater than ± 30 mV) create strong repulsive forces between droplets, preventing them from clumping together (agglomeration) due to van der Waals forces^59^. When the total attractive forces between droplets exceed the van der Waals force, the zeta potential increases, leading to particle dispersion and deagglomeration^60^. In this study, the formulations F1.1 (12.3 mV) and F1.2 (−10.6 mV) showed relatively low charge (Table 5), suggesting they may be less stable than the ideal ± 30 mV threshold. The stability of nanoemulsions F1.1 and F1.2 is governed by the interplay between droplet size, zeta potential, and PDI (Table 5). While F1.1 has a smaller average droplet size (106.3 nm) compared to F1.2 (114.1 nm), it is significantly less stable due to its high PDI (0.65). A PDI exceeding 0.5 indicates a broad size distribution, which triggers Ostwald ripening (condensation). In this process, smaller droplets coalesce into larger ones, releasing free energy and leading to thermodynamic instability^37,61,62^. Conversely, F1.2 is more stable because its lower PDI (0.33) indicates a more uniform distribution, despite having a slightly larger average droplet size and a lower zeta potential (−10.6 mV).

**Table 5:** Droplet size, Polydispersity index and Zeta potential of various nano-emulsions of two garlic extracts.

Formulation code	Emulsifier type	Droplet size (nm)	Polydispersity index PDI	Zeta Potential (mV)
F1.1	Tween 20	106.3^a^ ± 3.20	0.65 ± 0.020	12.3 ± 2.35
F1.2		114.1 ± 5.04	0.33 ± 0.012	−10.6 ± 1.65
F2.1	Tween 80	104.6 ± 3.07	0.42 ± 0.011	18.2 ± 2.50
F2.2		138.2 ± 5.73	0.36 ± 0.010	− 0.073 ± 1.62
F3.1	Tween 80 + Span80 blend	127.2 ± 11.14	0.40 ± 0.014	10.2 ± 2.03
F3.2		134.5 ± 3.98	0.35 ± 0.007	20.3 ± 6.80

^a^Each value represents the mean ± SD, and each measurement was performed after 16 weeks of storage.

F1.1, F2.1, and F3.1 code formulations of garlic ethanol extract.

F1.2, F2.2, and F3.2 code formulations of garlic ethyl acetate extract.

**Fig. 1:**
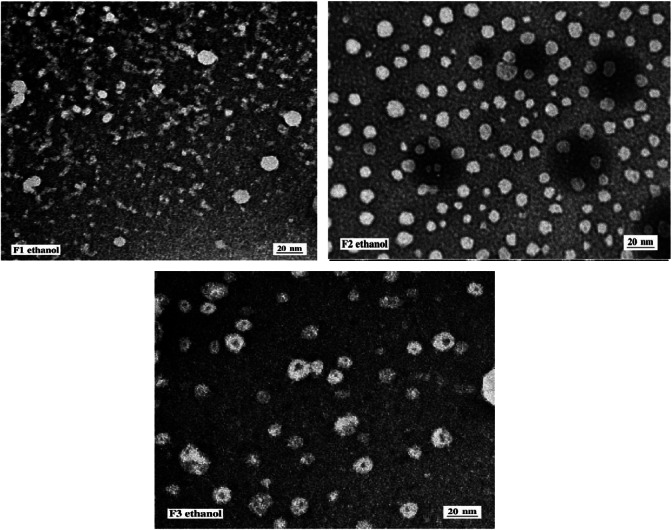
Transmission electron microscope of prepared nanoemulsions utilizing garlic oil extracted by ethanol. Nanoemulsions were developed using Tween 20 (F1), Tween 80 (F2), and a mixture of Tween 80 and Span80 (F3). Pl rearrange the images of figure 1 as those presented in figure 2 (two images above and one below)

**Fig. 2:**
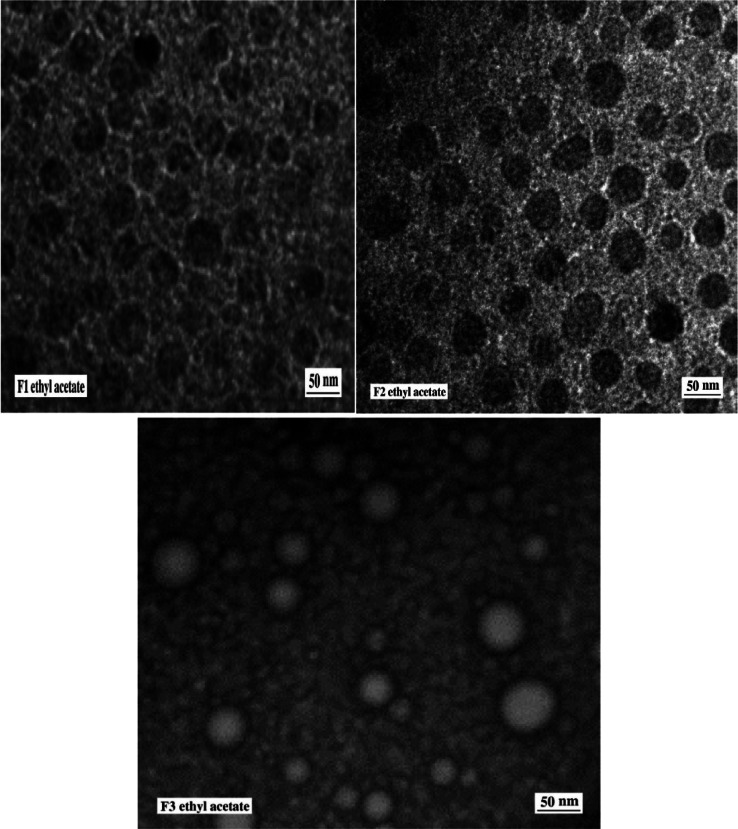
Transmission electron microscope of prepared nanoemulsions utilizing garlic oil extracted by ethyl acetate. Nanoemulsions were developed using Tween 20 (F1), Tween 80 (F2), and a mixture of Tween 80 and Span80 (F3).

Phase separation occurs when droplets condense into two distinct layers, often driven by Ostwald ripening^63^. In polydisperse systems, concentration gradients cause smaller droplets to shrink and larger ones to grow, eventually leading to structural collapse through creaming or sedimentation. To validate these stability predictions, electron microscopy is used to confirm the droplet size and morphology measured by light scattering. This ensures that the droplets are indeed spherical, a key assumption in sizing analysis and a critical factor in the formulation’s overall effectiveness. Transmission Electron Microscopy (TEM) confirmed that both nanoemulsion formulations, F1.1 and F1.2, consist of highly uniform, spherical droplets. The TEM results showed a median size of 20.04 nm for F1.1 and 27.74 nm for F1.2 (Figs. 1 and 2). These findings correlate closely with the hydrodynamic diameters previously measured by Dynamic Light Scattering (DLS), validating the accuracy of the sizing methods used.

### Method 2 (formulation code F2 using Tween 80)

This study utilized Tween 80 (a non-ionic surfactant) and citric acid to achieve micellization. While citric acid lowers the formulation’s viscosity and pH, it acts as a quality-enhancing additive that improves overall stability^64^. Specifically, this acidic environment makes the nanoemulsion highly durable for use in acidic food applications, such as beverages and dairy products^65^.

The formulations were developed utilizing Tween 80 as a surfactant, selected for its Hydrophilic-Lipophilic Balance (HLB) value of 15. The formulation process involved a high-energy ultrasonication method to break the primary emulsion into nanodroplets. By mixing garlic oil and Tween 80 at ratios between 1:1 and 1:1.2, then adding the mixture dropwise to distilled water containing 0.8% citric acid while the magnetic stirrer was continuously operated at 700 rpm for 15 min and applying ultrasonic treatment at 750 W for up to 35 min, the researchers created a stable system in a citric acid-distilled water base^37,38^.

The results of this study confirmed that 35 min of ultrasonication combined with specific oil-to-surfactant ratios produced nanoemulsions capable of surviving extreme stress tests, including centrifugation, heating, cooling, and freezing. However, stability is heavily influenced by pH levels. Rao and McClements^66^found that droplet formation and coagulation in the oil phase occurred at both high and low pH but were most pronounced in nanoemulsions with a pH between 5 and 6. While low pH can improve durability in specific applications (like acidic beverages), it can also accelerate a decrease in turbidity (clarity) during storage. Consistent with previous research, stability is most challenged at moderate pH levels (5–6), where droplet coagulation is most likely to occur^67^.

The obtained results indicated that the sonication time is the most critical factor for stability, whereas the oil-to-emulsifier ratio had a negligible impact. To create a consumer-friendly product, a low emulsifier concentration of 5% wt was selected, which was sufficient to coat and stabilize the newly formed droplets. The optimal formulation, designated as F2, utilizes a 1:1 (w/w) ratio of garlic oil extracted by both solvent types to Polysorbate 80 and 35 min of sonication. This specific configuration proved resilient against centrifugation and extreme temperature fluctuations (heating, refrigeration, and freezing).

The research demonstrates that the type of extraction solvent used for garlic oil directly influences the final particle size of the nanoemulsion. According to the study data, ethanol-extracted garlic oil produced smaller droplets (104.6 ± 3.07 nm) compared to ethyl acetate-extracted oil (138.2 ± 5.73 nm), as shown in Table 5. These results align with previous studies (Ziani et al.^68^,, Saberi et al.^69^,), which established that the chemical composition of the oil phase and the choice of emulsifier are primary determinants of colloidal properties. The consistent observation that droplet sizes remained above 100 nm suggests that the 1:1 ratio of oil-to-emulsifier created a substantial structural shell around the core, defining the final hydrodynamic diameter.

The investigation into the F2 formulation revealed that the choice of solvent—ethanol (F2.1) versus ethyl acetate (F2.2) significantly alters the electrical and physical characteristics of the nanodroplets. The higher the zeta potential value of the capsules, the less likely the droplets are to repel each other and agglomerate^70,71^. The polydispersity indices of all formulation samples were close to or below 0.4, which is generally within the acceptable range and indicates a uniform particle size. F2.1 formulation is considered more robust because its zeta potential (18.2 mV) provides sufficient electrical repulsion to keep droplets apart. According to zeta potential, significant agglomeration occurs between 3 and 5 mV, while high stability and minimal agglomeration are observed between 5 and 15 mV^72^. Despite a near-zero zeta potential (−0.0727 mV), F2.2 remains stable. This is attributed to steric stabilization, where the non-ionic surfactant Tween 80 creates a dense physical “coating” around the droplets, mechanically blocking them from merging^70^.

The morphology of the F2.1 nanoemulsion was characterized using Transmission Electron Microscopy (TEM), confirming that the garlic oil is encapsulated in symmetrical, spherical particles. TEM analysis revealed an average particle size of approximately 45.61 nm, which is significantly smaller than the sizes typically reported via Dynamic Light Scattering (DLS). DSL measures the hydrodynamic diameter of particles in a hydrated, dissolved state. This includes the oil core, the surfactant layer, and the associated water shell, often resulting in larger recorded values. On the other hand, TEM measures the actual physical dimensions of the dried particle core under an ultra-high vacuum.

### Method 3 (formulation code F3 using Tween 80 and Span 80 blend)

The Hydrophilic-Lipophilic Balance (HLB) is a critical metric for selecting an emulsifier that matches the amphiphilic nature of the oil phase. Based on the methodology of Liu et al.^73^, maximum stability is achieved when the surfactant system’s HLB matches the “required HLB” of the oil. To reach this target for garlic oil, a surfactant mixture (*Smix*) was created by blending Tween 80 (high HLB) and Span 80 (low HLB). This allowed the researchers to fine-tune the *Smix* value across a range from 7 to 15 to identify the most stable formulation. The HLB value of *Smix* can be calculated using the equation:

$${\rm{HL}}{{\rm{B}}_{\rm{m}}} = {\rm{ HL}}{{\rm{B}}_{\rm{t}}} \times {\rm{ t }} + ~{\rm{HL}}{{\rm{B}}_{\rm{s}}} \times {\rm{ s}}$$

Where HLBt and HLBs are the respective HLB values of Tween 80 and Span 80, respectively, and HLBm represents the HLB value of *Smix*. The variables t and s represent the mixing ratios of Tween 80 and Span 80, respectively. To identify the ideal surfactant environment, researchers tested a range of surfactant mixtures (*Smix*) by blending Span 80 and Tween 80 in nine different proportions, resulting in HLB values ranging from 7 to 15. Following the principles established by Cherkas et al.^74^, a nanoemulsion is most stable when the HLB of the emulsifier matches the HLB of the oil phase. The study found a non-linear relationship between HLB and particle size: as the HLB increased from 7 to 14, the particle size reached its minimum average value. Based on these results and supporting evidence from Long et al.^39^, HLB 14 was determined to be the optimal value for garlic oil, providing the highest level of interfacial stabilization.

To achieve the ideal HLB of 14, the study utilized a surfactant mixture (*Smix*) of Tween 80 and Span 80 at a 9:1 ratio (Table 4). This specific combination yielded highly transparent nanoemulsions that remained stable for 16 weeks at room temperature with no signs of aggregation, sedimentation, or creaming and the smallest average particle sizes, 127.2 nm for ethanol-extracted garlic oil and 134.5 nm for ethyl acetate-extracted garlic oil (Table 5). Generally, the nanoemulsion particle sizes can range from 20 to 200 nm^75^.When the HLB value of the surfactant combination matches the HLB value of the oil, nanoemulsions with smaller droplet sizes can be produced^54,55^. Final optimized process combined 5.5% garlic oil with 10% *Smix*, processed via ultrasonic probe at 80% output for 5 min.

Particle homogeneity is quantified by the particle PDI, which ranges from 0 to 1; values lower than 0.5 indicate homogeneous particles. Table 5 showed the PDI was 0.40 and 0.35 and the average zeta potential was 0.2 and 20.3, for the nano formulation contained garlic oil extracted from ethanol extract and ethyl acetate extract, respectively. Transmission electron microscopy (TEM) was employed to examine the resulting garlic oil nano-emulsion. Because TEM was performed in dry conditions and the obtained droplet sizes tend to shrink, losing their initial shape and size^76^, in our study, it was discovered that the nano-emulsion droplet sizes of F3.1 (35.468 nm) and F3.2 (44.745 nm) were smaller than their mean calculated by the DLS, as shown in Table 5.

The uniformity of the particles is quantified by the particle PDI, which has a value between 0 and 1, and a value less than 0.5 indicates uniform particles. The PDIs of the nanoformulations containing garlic oil extracted with ethanol and ethyl acetate were 0.40 and 0.35, respectively, and the average zeta potentials were 10.2 mV and 20.3 mV, respectively (Table 5). Transmission electron microscopy (TEM) was used to observe the produced garlic oil nanoemulsions. The size of the obtained droplets tends to shrink and lose their initial shape and size^76^. In this study, it was confirmed from Table 5 that the sizes of the F3.1 (35.468 nm) and F3.2 (44.745 nm) nanoemulsion droplets (Figs. 1 and 2) were smaller than the average values calculated by DLS.

For the preparation of nanoemulsions, three emulsifiers were selected based on their HLB values. Formulations were developed using different emulsifier-to-oil ratios using each of the three emulsifiers. These formulations were selected based on their PDI values, droplet size, and overall appearance. While creating a transparent formulation was challenging, we were able to obtain formulations with appropriate droplet sizes and PDI values. Depending on the preparation method, various droplet size distributions can be obtained, demonstrating that different preparation methods can affect the viscosity of nanoemulsions (Table 5). Conventional emulsions and microemulsions, ranging in size from 20 to 500 nm, are classified as miniemulsions, ultrafine emulsions, transparent emulsions, nanoemulsions, and submicron emulsions. Nanoemulsions exhibit a translucent and continuous form due to their tiny droplet sizes. They offer excellent stability due to continuous Brownian motion, which prevents creaming and sedimentation^77^.

The evaluation of nanoemulsions is largely dependent on droplet size. As particle size decreases, the permeation surface area increases. The PDI is a measure of the droplet size distribution within a system. The PDI indicates the uniformity and stability of droplet sizes within an emulsion. Furthermore, the charge distribution throughout the system can be expressed as the zeta potential. Each particle within the system has a unique surface charge. A uniform surface charge distribution is essential for a homogeneous formulation that prevents aggregation and stabilizes the nanoemulsion. Generally, higher PDI (< 0.2) and zeta potential values indicate better nanoemulsion stability^78^.

Garlic oil formulations prepared using ethanol extraction, incorporating Tween 80 as a surfactant along with citric acid had lower transparency than formulations using Tween 20 or a blend of Tween 80 and Span 80. This reduced clarity posed challenges in the manufacturing process. Although Tween 80 alone did not achieve clarity, it achieved the smallest droplet size (104.6 nm) and the highest zeta potential (18.2 mV), showing results similar to those of the other two preparing methods. Based on the three parameters characterizing nanoformulations, it is believed that selecting a formulation using Tween 20 or a blend of Tween 80 and Span 80 for garlic oil formulations extracted using ethyl acetate solvent will yield results similar to those of Tween 80 alone. Meanwhile, the blend of Tween 80 and Span 80 exhibited higher zeta potential (20.3 mV) and PDI (0.35), which are expected to help extend the shelf life of the formulation.

Furthermore, it can be concluded that the properties of the encapsulated oil, such as interfacial tension and viscosity, significantly influence the persistence of nanoemulsions^57^. In addition, as the viscosity of the dispersed phase increased, the average particle diameter significantly increased^79^. This can be explained by the fact that nanoemulsions developed from garlic oil extracted with ethyl acetate always had larger droplet sizes, regardless of the type of surfactant used in the formulation.

### Comparative nematicidal activity of garlic extract nanoemulsion formulations against tomato root-knot nematode (*M. incognita*) under greenhouse conditions

In this study, we compared six nanoemulsion formulations of garlic oil extracted with ethanol (F1.1, F2.1, F3.1) and ethyl acetate (F1.2, F2.2, F3.2) with cadusafos and oxamyl to evaluate their effectiveness in controlling *M. incognita* on tomatoes in a pot experiment (Table 6). Depending on the solvent used to extract the garlic oil and prepare their nano-emulsions, resulting in varying potencies against *M. incognita*. In general, our investigation clearly demonstrated that all tested nanoemulsions and commercial nematicides significantly suppressed nematode parameters in terms of root galls and J2 numbers in the soil. The extent to which nematode reduction, however, varied among the treatments. Compared to the inoculated control, all tested treatments significantly reduced the number of root galls. However, F2.1 and F2.2 had the highest reduction in root galls (87.10% and 87.56%, respectively), followed by F1.1 (74.19%), F3.1 (73.04%), and F1.2 (62.67%). It is also important to note that there was no significant difference in this regard between the F1.1 and F3.1 treatment and oxamyl (71.66%). Likewise, there was no significant difference between F1.2 and the nematicide cadusafos (55.76%). In fact, it is noteworthy that both F2.1 and F2.2 were significantly more effective at reducing root gall numbers than the nematicides tested, for which there was no significant difference (Table 6).

**Table 6:** *In vivo* effectiveness of six garlic oil nano-emulsions against the root-knot nematode *Meloidogyne incognita* on tomato.

Treatments	Emulsifier type	*Galls/plant	% Reduction	* J2/250 g soil	% Reduction
F1.1 F1.2	Tween 20	186.7 ± 3.333 ^ef^ 270.0 ± 11.547 ^cd^	74.19 62.67	33.3 ± 3.016 ^h^ 116.6 ± 0.746 ^gf^	98.62 95.18
F2.1 F2.2	Tween 80	93.3 ± 3.333 ^g^ 90.0 ± 5.773 ^g^	87.10 87.56	50.0 ± 3.763 ^h^ 66.6 ± 3.016 ^gh^	97.93 97.24
F3.1 F3.2	Tween 80 + Span80 blend	195.0 ± 8.660 ^ef^ 436.7 ± 31.797 ^b^	73.04 39.62	366.6 ± 0.430 ^d^ 1683.3 ± 0.728^b^	84.83 30.34
Oxamyl	-	205.0 ± 18.027 ^e^	71.66	180.0 ± 0.288 ^ef^	92.55
Cadusafos	-	320.0 ± 15.275 ^c^	55.76	390.0 ± 0.291 ^dc^	83.86
Inoculated control	-	723.3 ± 54.569 ^a^	-	2416.6 ± 0.613^a^	-

F1.1, F2.1, and F3.1 code formulations of garlic ethanol extract.

F1.2, F2.2, and F3.2 code formulations of garlic ethyl acetate extract.

*Each value is a mean ± SD of six replicates. Means within the same column and exposure times followed by the same letter are not significantly different according to one-way ANOVA with Duncan tests *p* < 0.05.

The data also clarified that all tested treatments substantially decreased the soil’s J2 populations compared to the control. The greatest reduction was noted with F1.1 (98.62%), followed by F2.1 and F2.2 (97.93% and 97.24%, respectively), F1.2 (95.18%) and then F3.1 (84.83%). However, it did not cause a noticeable decrease in J2 compared to ethyl acetate extracts that were formulated by using Tween 20 (F1.2) or Tween 80 (F2.2). Similarly, F1.2 and oxamyl (92.55%) did not differ significantly. It was also observed that the ethanolic extract prepared by a combination of Tween 80 and Span 80 (F3. 1) and cadusafos (83. 86%) did not differ significantly. Compared to the nematicides tested, all ethyl acetate and ethanol garlic oil formulations employing either Tween 20 (F1. 1 and F1. 2) or Tween 80 (F2. 1 and F2. 2) demonstrated a higher reduction in J2 (Table 6).

The transition from conventional botanical extracts to Nano-emulsion (NE) systems represents a paradigm shift in the management of plant diseases, including PPNs^80,81^. In our study, soil drenching with nanoemulsion-based-ethanol and -based-ethyl acetate formulations containing garlic oil extract demonstrated nematicidal activity against *M. incognita* and promoted tomato growth compared to untreated controls, however, the ethanol-based formulation was more effective than the ethyl acetate one. Bar et al.^13^ reported that the extraction solvent can affect the types of compounds extractable from garlic, resulting in differences in biological activity. Likewise, recent studies on eggplants^32^ and rice^33^ demonstrated significant reductions in nematode infestations and improved crop growth parameters, emphasizing the potential of garlic oil nanoemulsions as botanical nematicides in integrated pest management and sustainable agriculture.

The nematicidal efficacy of garlic oil extract in nano-emulsified form may be due to several ways, including their high content of sulfur-containing volatile organic compounds (e.g. diallyl polysulfides)^21,30^. They physically facilitate the penetration of nano-droplets through the nematode cuticle and the protective layer of eggs by improving kinetic stability and surface reactivity. Moreover, they reduce the rapid evaporation of sulfur components from the oil and increase the oil’s movement through soil pores, allowing it to reach the rhizosphere where nematodes gather. Furthermore, these compounds are known to affect nematode physiology by influencing their populations’ mobility, growth and reproduction, ion uptake, permeability, enzymatic activity, cell division, redox reactions, and react with the thiol group of enzymes essential in the nematode metabolism, ultimately leading to paralysis and death^82–84^. According to Wang et al.^85^, the bio-nematicide dimethyl disulfide contained in garlic essential oil exerts its unique biological effects on nematodes through two distinct mechanisms; direct contact *via* penetrates the nematode’s cuticle and damaging the structural integrity of the cell membranes of the body wall and muscle tissue. It also acts as an uncoupler of oxidative phosphorylation pathway. As a fumigant, dimethyl disulfide enters the body *via* the olfactory respiratory oxygen exchange pathway, generates more sustained calcium signals, modulates neurotransmitter release, and ultimately targets cytochrome c oxidase or NADH dehydrogenase, leading to the death of the nematode. Extracts of *A. sativum*have been found to enhance lignification and promote callose deposition in root tissues, effectively obstructing the invasion and development of nematodes^86^. Recently, researchers have reported that nanoemulsified garlic oil may act as an elicitor, triggering the plant’s own defense genes (e.g., pathogenesis-related proteins) and promoting the expression of defense-related enzymes such as peroxidase and polyphenol oxidase^9,87^.

Table 7 presents the effects of the tested treatments on tomato growth characteristics, specifically focusing on the length and weight of the shoot and root systems. The results indicated that the smallest plant growth characteristics was observed in the control group inoculated with nematodes. All developed treatments (F1.1, F1.2, F2.1, F2.2, F3.1, and F3.2) notably enhanced both shoot and root length compared to the inoculated control. However, no notable differences were detected across these treatments. Interestingly, the results revealed that the effects of the developed treatments were statistically comparable to those of the two nematicides, oxamyl and cadusafos. When considering shoot fresh weight, treatments involving oil extracted with ethyl acetate (F1.2) and ethanol (F2.1), as well as oxamyl, caused notable increases relative to the inoculated control, except for F2.2 and F3.2. No significant difference was detected between F1.1, F3.1, and cadusafos compared to the inoculated control, except for oxamyl. Regarding root fresh weight, all treatments, including the traditional nematicides, showed no significant difference from the inoculated control. However, treatments F3.1 and F3.2 resulted in a significant reduction in root fresh weight. This reduction was comparable to the decreases observed with oxamyl and cadusafos (Table 7). These findings align with earlier research demonstrating no adverse effects on plants when garlic oil extracts, garlic essential oil vapor or its sulfur-containing volatile organic compounds are used^29,88^.

**Table 7:** Growth response of tomato plants, infested with *M. incognita*, to six garlic oil nano-emulsions under greenhouse conditions.

Treatment	Emulsifier type	Growth indices			
		Shoot		Root	
		Length (cm)	Fresh weight (g)	Length (cm)	Fresh weight (g)
F1.1	Tween 20	25.7 ± 1.856^ab^	5.83 ± 0.1660^d^	15.0 ± 1.000^ab^	1.9 ± 0.100^bc^
F1.2		25.3 ± 0.333^ab^	8.67 ± 0.440^a^	13.4 ± 0.500^abc^	2.4 ± 0.600^bc^
F2.1	Tween 80	23.3 ± 2.906^bc^	7.67 ± 0.166^b^	11.8 ± 1.922^bc^	1.6 ± 0.288^bc^
F2.2		24.3 ± 0.882^abc^	5.00 ± 0.288^ef^	15.3 ± 1.333^a^	1.8 ± 0.166^bc^
F3.1	Tween 80 + Span80 blend	27.2 ± 0.833^ab^	6.2 ± 0.600^d^	13.1 ± 1.166^abc^	1.3 ± 0.166^c^
F3.2		25.3 ± 0.666^ab^	4.8 ± 0.166^f^	15.3 ± 0.666^a^	2.2 ± 0.166^bc^
Oxamyl		27.3 ± 0.666^a^	8.7 ± 0.1666^a^	14.6 ± 0.666^abc^	2.0 ± 0.288^bc^
Cadusafos		25.3 ± 0.666^ab^	6.8 ± 0.166^bcd^	15.3 ± 0.333^a^	2.1 ± 0.166^bc^
Uninoculated control		20.9 ± 0.829^c^	7.3 ± 0.145^bc^	11.7 ± 0.333^c^	4.0 ± 0.577^a^
Inoculated control		15.3 ± 0.333^d^	6.0 ± 0.288^d^	8.8 ± 0.166^d^	2.8 ± 0.440^b^

F1.1, F2.1, and F3.1 code formulations of garlic ethanol extract.

F1.2, F2.2, and F3.2 code formulations of garlic ethyl acetate extract.

*Each value is a mean of six replicates. Means within the same column and exposure times followed by the same letter are not significantly different according to one-way ANOVA with LSD and Duncan tests *p* < 0.05.

Soil drenching with plant extracts/oils and their metabolites, are powerful eco-friendly tools that act as natural fertilizers, promoting plant growth, nutrient uptake, and stress tolerance, and function as nematicides to control plant-parasitic nematodes, offering sustainable alternatives to chemical nematicides for soil and crop health^89^. Accordingly, our study showed that GEO-NEs with the main components diallyl polysulfides demonstrated remarkable *in vivo* nematicidal efficacy against *M. incognita* and significantly enhanced tomato growth performance. These results are comparable to those obtained using garlic extract or essential oil that have been shown to be effective in controlling *M. incognita*^19,28–30^. Khairan et al^20^. tested the efficacy of garlic aqueous extract (AGE), methanol extract (MGE), ethyl acetate extract (EAGE), and n-hexane extract (HGE) against the root-knot nematodes (*Meloidogyne* sp.) in a laboratory setting. The list below shows the level of activity of garlic extracts against *Meloidogyne* sp.in the order of EAGE > AGE > MGE > HGE. In greenhouses, both aqueous and ethanolic extracts of garlic improved tomato growth parameters and reduced *M. incognita*indices. However, the ethanolic extract showed the greatest reduction in the number of 2nd jeveniles (97.8%) and galls (94.3%), compared to the aqueous extract, which was 82.4% and 90.3%, respectively, indicating a promising use as an eco-friendly strategy for root-knot nematode control^31^. An aqueous garlic extract also improved the physiology and growth of tomato seedlings when used as foliar feeding and/or fertigation. Plant length, leaf area, stem size, and fresh and dry weight increased significantly as a result of the treatments^90^. Recently, the application of GEO-NE as a soil fertilizer at a rate of 10 ml per plant significantly improved the growth performance of eggplants compared to untreated inoculated plants^32^.

## Conclusion

This study stands out for its comprehensive analysis of six developed and characterized nanoemulsions, followed by their screening to identify the most effective formulation. Unlike previous research on garlic-extract-based treatments, this work adopts a unique approach, particularly in its selection of solvents and its comparative performance against *M. incognita*. Notably, the nanoemulsions were applied as a soil drench rather than being limited to *in vitro* testing. The evaluations, conducted in pots, further enhance the study’s robustness and represent a significant step towards practical field applications. Analysis of garlic oil extracts by GC-MS revealed that sulfur-containing compounds varied depending on the type of solvent used, which accounts for nematicidal efficacy. Ultrasonic emulsification using specific surfactants (e.g., Tween 20, Tween 80, and Tween 80/Span 80 blend) proved to be an effective and practical method for developing nanoemulsions from extracted garlic oil. Specifically, the PDI and particle size of GONE significantly decreased with increasing sonication time. Furthermore, excellent storage stability was observed for up to 16 weeks. The resulting nanoemulsions were characterized by very small droplets (≤ 138 nm), PDI (≤ 0.65) and zeta potential values in the range of 10.2–20.3 mV, which improved their ability to penetrate the nematode cuticle and disperse in the soil. These promising findings suggest that nanoemulsions can effectively manage this nematode species by reducing the formation of root galls and 2nd juveniles in the soil and promoting tomato growth, particularly the Tween 20 and Tween 80 formulations, both of which were ethanol-based and demonstrated superior nematicidal effects compared to ethyl acetate extract as well as the tested chemical nematicides; cadusafos and oxamyl. Overall, the garlic oil nanoemulsion formulations developed in our study appear to be promising candidates for the development of novel and green nematicide formulations for managing *M. incognita* associated with tomatoes in sustainable agriculture, while also highlighting the need for additional field validation and their application for effective nematode control in organic farming, as well as their side effects on non-target organisms.

## Data Availability

All data analyzed during this study are included in this article.The raw data that support the findings of this study are available on request from the corresponding author.
